# Investigating COX-2 and 5-LOX Enzyme-Related Anti-Inflammatory and Antioxidant Activities and Phytochemical Features of *Scutellaria salviifolia* Benth

**DOI:** 10.3390/ijms26125608

**Published:** 2025-06-11

**Authors:** Gülsüm Metkin, İpek Süntar, Fatma Sezer Şenol Deniz, Osman Tugay, Mustafa Demiralp, Valeria Pittalà

**Affiliations:** 1 Department of Pharmacognosy, Faculty of Pharmacy, Selcuk University, Konya 42130, Türkiye; gulsum.bosdanci@selcuk.edu.tr; 2 Department of Pharmacognosy, Faculty of Pharmacy, Gazi University, Ankara 06330, Türkiye; fssenol@gazi.edu.tr; 3 Department of Pharmaceutical Botany, Faculty of Pharmacy, Selcuk University, Konya 42250, Türkiye; otugay@selcuk.edu.tr; 4 Advanced Technology Application and Research Center, Sivas Cumhuriyet University, Sivas 58140, Türkiye; mdemiralp@cumhuriyet.edu.tr; 5 Department of Drug and Health Sciences, University of Catania, 95125 Catania, Italy

**Keywords:** anti-inflammatory, antioxidant, enzyme inhibition, Lamiaceae, *Scutellaria salviifolia*, 5-LOX, COX-2

## Abstract

*Scutellaria* species are widely utilized and have demonstrated diverse biological effects for various diseases, both globally and in traditional Chinese medicine, due to the presence of bioactive compounds with unique structures. This study was conducted to reveal the in vitro effects and phytochemical properties of *Scutellaria salviifolia* Benth., an endemic species of Türkiye. The inhibitory effects of methanol extracts prepared separately from the aerial and root parts of *S. salviifolia* on the COX-2 and 5-LOX enzymes and their DPPH and ABTS radical scavenging activities were evaluated using in vitro methods. Additionally, the phenolic compounds of the extracts were compared based on Q-TOF LC/MS analysis. The extracts of *S. salviifolia* exhibited a high inhibitory effect on COX-2 enzyme activity, comparable to that of celecoxib. Still, they showed no significant effects in the 5-LOX enzyme inhibition assay. In the antioxidant activity assays, the percentage of inhibitory effects of both extracts against DPPH and ABTS were similar. A total of 29 and 27 compounds were detected in the aerial part and root extracts, respectively. Among the identified compounds, 18 were common to both the aerial part and root extracts. *S. salviifolia* may serve as a valuable alternative to the most well-known *Scutellaria* species, including *S. baicalensis* and *S. barbata*.

## 1. Introduction

Species belonging to the Lamiaceae family are plants with therapeutic properties, widely utilized in traditional medicine worldwide and frequently investigated in ethnopharmacological studies. In the flora of Türkiye, the Lamiaceae family ranks third in taxonomic diversity, with 48 genera and 782 taxa, 44% of which are endemic [1]. *Scutellaria* L., a significant genus within this family, is native to Europe, the United States, and East Asia. There are 350 recognized species within this genus, including 17 species and 39 taxa found in Türkiye [2,3].

Most *Scutellaria* species are annual or perennial herbs ranging from 5 cm to 1 m in height, although a few are subshrubs. These species feature four-angled stems, opposite leaves, and bilabiate flowers. The genus is most easily identified by the distinctive shield-shaped calyx [2].

Various species of the *Scutellaria* genus have been employed in traditional herbal medicine for the treatment of numerous ailments, including cancers, liver and stomach disorders, respiratory system diseases, neurological and cardiovascular conditions, and infections, since ancient times [4,5,6,7,8,9]. In East Asia, certain *Scutellaria* species have been extensively utilized in traditional medicine, particularly in China, Korea, and Japan, for their anti-inflammatory, antiviral, sedative, antithrombotic, and antioxidant properties. Radix *Scutellariae* is officially recognized in the Chinese Pharmacopoeia (2015), British Pharmacopoeia (2018), and European Pharmacopoeia (9.0) [6]. The most well-known species of this genus are *Scutellaria baicalensis* Georgi and *Scutellaria barbata* D.Don. *S. baicalensis* is primarily used in the treatment of inflammatory diseases such as dermatitis, gingivitis, and ulcers due to its anti-inflammatory properties, while *S. barbata* is utilized for its anticancer effects [10,11,12]. Radix *Scutellariae baicalensis* (HUANG QIN) and Herba *Scutellariae barbatae* (BAN-ZHI-LIAN) are registered in the Pharmacopoeia of the People’s Republic of China and Japan [13,14].

*Scutellaria* spp. are referred to as “kaside”, “korku otu”, “sancı otu”, and “şimşek otu” and are most commonly employed as sedatives, hemostatic agents, wound healers, and tonics in Türkiye [15,16]. There are also records of the traditional use of *S. salviifolia* as an astringent, wound-healer, carminative, and relaxant for stomach ailments [16,17,18,19,20,21,22,23,24,25,26]. Recent research on *S. salviifolia* has explored its potential anti-inflammatory effects in the treatment of gastric disorders, suggesting it may offer therapeutic benefits for managing gastrointestinal inflammation [27].

Phytochemical analyses of *Scutellaria* species have identified the presence of flavonoids, iridoid glycosides, phenylethanoid glycosides, diterpenes, triterpenes, alkaloids, and essential oil compounds [2,28]. Extracts prepared from various parts of these species, as well as isolated compounds, have demonstrated a range of biological activities, including hepatoprotective [2], antiangiogenic [29], neuroprotective [30], anticonvulsant [31], antioxidant, antimicrobial [32], antiviral [33], antihyperglycemic [34], and antitumor effects against leukemia, colon cancer, hepatoma, lung cancer, skin cancer, and gynecological tumor cells [35]. More specifically, the main primary flavonoids including baicalin, baicalein, wogonin, wogonoside, and scutellarin were demonstrated to display pharmacological properties, including anti-inflammatory, antioxidant, anti-tumor, antibacterial, antiviral, hepatoprotective, and neuroprotective activities [36,37].

Given that oxidation and inflammation are underlying causes of the pathogenesis of these chronic diseases, and considering the potential enzyme inhibition activities regarding the anti-inflammatory and antioxidant properties of natural compounds within this genus, it is essential to explore additional *Scutellaria* species as alternative sources of therapeutic agents. Consequently, the present study was undertaken to evaluate the in vitro enzyme inhibitory activities of *Scutellaria salviifolia* Benth., an endemic species of Türkiye, along with its phytochemical composition. The current study is the first to comparatively investigate the inhibitory effects of the extracts prepared from the aerial parts and roots of *S. salviifolia* on the 5-lipoxygenase (5-LOX) and cyclooxygenase (COX)-2 enzymes and to compare their respective compositions.

## 2. Results

### 2.1. Enzyme Inhibition Assays

Enzyme inhibition tests were conducted using kits containing human recombinant 5-LOX and COX-2 enzymes and were performed with methanol extracts prepared from the roots and aerial parts of *S. salviifolia.* All enzyme inhibition assays were performed in triplicate. The final concentration of the extracts was 400 µg/mL for all enzyme inhibition tests [38].

**Figure 1:**
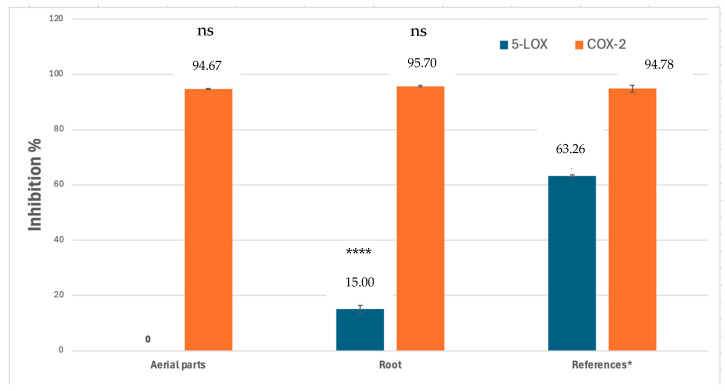
5-LOX and COX-2 enzyme inhibition values of *S. salviifolia* extracts at 400 µg/mL final concentration (* Zileuton for 5-LOX and celecoxib for COX-2, ns: not significant, **** *p* < 0.0001).

No significant inhibitory activity was observed for 5-LOX with the extracts. However, in the COX-2 enzyme inhibition assay, both the aerial part and root extracts demonstrated high inhibition values comparable to the reference. The highest inhibitory effect was observed with the root extract (95.70 ± 0.35%), while the inhibition values of the aerial part extract (94.671 ± 0.15%) and celecoxib (94.78 ± 1.16%) were found to be very similar. The enzyme inhibition values for the extracts and references are presented in Figure 1.

### 2.2. Antioxidant Activity Assays

The antioxidant effects of methanolic extracts prepared from the aerial and roots of *S. salviifolia* were evaluated using ABTS and DPPH radical scavenging activity determination assays. All antioxidant activity analyses were performed in four replicates, and the results are expressed as the mean ± standard deviation (S.D.). The stock concentration of the extracts was 2 mg/mL in both assays.

Both the aerial parts and root extracts demonstrated similar and high radical scavenging activity compared to the reference compounds in both the ABTS and DPPH assays. In the ABTS assay, the aerial part extract exhibited 89.2 ± 0.03% radical scavenging activity, while the root extract showed 88.6 ± 0.88% radical scavenging activity. These values were found to be close (not significant compared to BHT) to the percentage inhibition value of BHT, the reference compound (89.5 ± 0.26%). In the DPPH assay, the aerial part extract showed 86.8 ± 0.28% (*p* < 0.1) radical scavenging activity, and the root extract showed 87.9 ± 0.36% (not significant compared to quercetin) radical scavenging activity. These values were comparable to the percentage inhibition value of quercetin, the reference compound (87.4 ± 0.32%).

According to the IC_50_ values obtained from the ABTS and DPPH radical scavenging activity assays, the aerial part extract exhibited the highest activity after the reference compounds in the ABTS assay (28.77 ± 0.20). In the DPPH assay, the aerial part and root extracts showed very similar effects, with IC_50_ values of 40.7 ± 1.15 and 40.1 ± 0.81, respectively. The ABTS and DPPH radical scavenging effects and IC_50_ values for the *S. salviifolia* extracts and references are presented in Table 1.

### 2.3. Phytochemical Analysis

**Figure 2:**
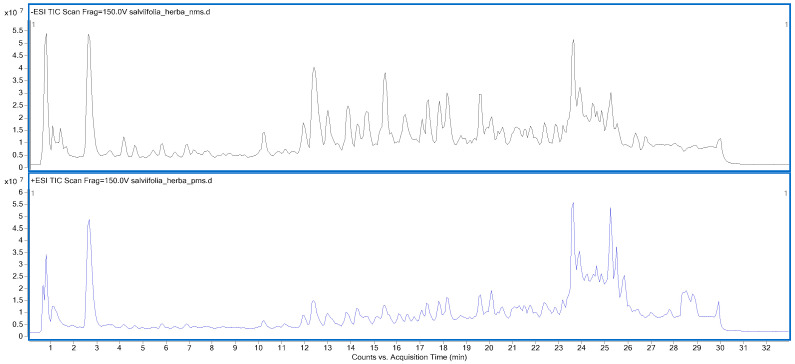
Total ion chromatogram (TIC) of *S. salviifolia* aerial part extract in negative (**above**) and positive (**below**) ionization modes.

**Figure 3:**
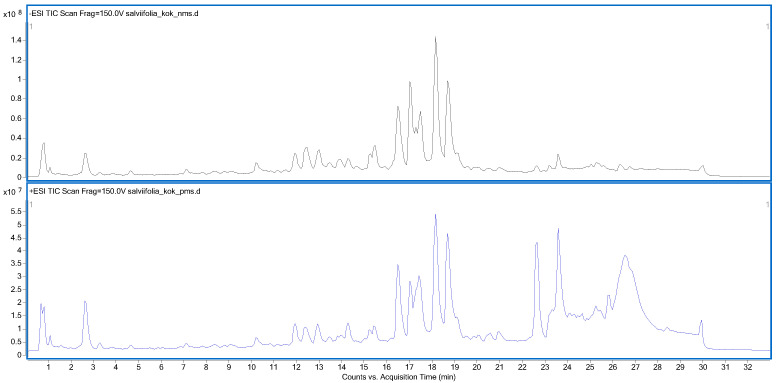
Total ion chromatogram (TIC) of *S. salviifolia* root extract in negative (**above**) and positive (**below**) ionization modes.

In the present study, the Q-TOF LC/MS system was employed to determine the phenolic composition of the *S. salviifolia* aerial part and root extracts. The tentative identification of compounds was achieved by comparing the precursor and product ions (formed as a result of different applied collision energies) with data reported in the literature and with information available in the MassHunter Metlin PCDL Manager (B.07.00) and PubChem databases. A total of 29 and 27 compounds were detected in the aerial part and root extracts, respectively, in both negative and positive ionization modes (Table 2). Among the identified compounds, 18 were found to be common to both the aerial part and root extracts. All identified compounds were confirmed based on both the generated precursor ions and their corresponding fragment ions, with a mass error of less than 5 ppm. Total ion chromatograms resulting from the analyses are provided in Figure 2 and Figure 3.

## 3. Discussion

Chronic inflammation and oxidative stress affect several physiological systems and are a prevalent underlying cause of a variety of disorders. Although inflammation is necessary to combat infections, excessive or protracted inflammation can lead to the onset and advancement of many chronic illnesses. To effectively treat conditions such as cancer, autoimmune diseases, metabolic disorders, respiratory diseases, cardiovascular diseases, and others, it is crucial to manage inflammation [39].

Arachidonic acid (AA) and its derivatives, prostaglandins (PGs) and leukotrienes (LTs), are crucial intracellular signaling molecules that regulate pain and inflammation processes. AA, a 20-carbon polyunsaturated fatty acid, is released from phospholipids in the cell membrane by the enzyme phospholipase A2. This fatty acid is further metabolized by the COX and 5-LOX enzymes, resulting in the formation of prostaglandins and leukotrienes. COX catalyzes the conversion of AA to prostaglandin G2 (PGG2), which is then converted to PGH2. PGH2 is further transformed into various prostaglandins (e.g., PGE2, PGD2, PGF2α, PGI2) and thromboxane A2, which play essential roles in fever, pain, swelling, inflammation, and platelet aggregation. These processes are central to the regulation of inflammation and pain responses [8,40]. COX-1 is an enzyme that is constitutively expressed in most tissues and is involved in the production of prostaglandins that maintain normal physiological functions, such as protecting the gastric mucosa, supporting kidney function, and regulating platelet aggregation. COX-2, on the other hand, is responsible for the time-dependent and localized production of prostaglandins, particularly at inflammatory sites. The overexpression of COX-2 has been associated with various diseases, including chronic inflammatory diseases such as rheumatoid arthritis, neurodegenerative disorders such as Parkinson’s disease, and several types of cancer [41,42,43]. The inhibition of COX-2 plays a critical role in controlling inflammation, as this enzyme is primarily responsible for the production of pro-inflammatory prostaglandins at the sites of injury or infection. On the other hand, to enhance the therapeutic potential of COX-2 inhibition, it is crucial to experimentally assess COX-1 activity through in silico, in vitro, or in vivo approaches to ensure true selectivity and minimize off-target effects.

5-LOX plays a critical role in the metabolism of AA, particularly in the biosynthesis of leukotrienes by oxidizing AA to 5-hydroperoxyeicosatetraenoic acid (5-HPETE) [44]. Leukotrienes, including LTB4, LTC4, LTD4, and LTE4, play a crucial role in regulating the body’s innate immune response. These mediators, especially LTB4, have been implicated in chronic inflammatory conditions such as rheumatoid arthritis, osteoporosis, and asthma [45,46,47,48]. Therefore, inhibiting COX-2 and 5-LOX and their downstream products is often targeted in the development of anti-inflammatory therapies.

The development and progression of many diseases are also influenced by oxidative stress, which is defined as an imbalance between the body’s antioxidants and free radicals, specifically reactive oxygen species (ROS). Free radicals are highly reactive chemicals that can damage DNA, proteins, and lipids in cells, leading to dysfunction and disease. ROS influence cellular processes, including growth, differentiation, progression, and cell death. Excessive ROS production contributes to the pathogenesis of numerous conditions, including arthritis, cancer, diabetes, atherosclerosis, ischemia, and dysfunctions in the immune and endocrine systems. Antioxidants mitigate the accumulation of ROS and reduce their harmful effects, thus playing a protective role in cellular health [49]. Excessive ROS production can oxidize biomolecules, structurally alter proteins and genes, and trigger signaling pathways that lead to the onset and progression of inflammatory diseases. These processes activate pro-inflammatory transcription factors and genes, leading to inflammation, cytokine secretion, and the recruitment of immune cells to the site of oxidative stress or infection. Consequently, increased ROS production by immune cells at the site of inflammation leads to further oxidative stress and tissue damage [50].

The present study focused on *S. salviifolia,* with the potential to display anti-inflammatory and antioxidant activities. The effects of methanol extracts prepared from the aerial and root parts of the plant were evaluated through in vitro methods using COX-2 and 5-LOX test kits, along with DPPH and ABTS assays. We found that *S. salviifolia* inhibited COX-2 but did not inhibit LOX-5, suggesting that it is selectively targeting the COX pathway without affecting the LOX pathway, specifically the LOX-5 isoform. The normal function of leukotrienes, which may be crucial in other aspects of immune function and inflammatory responses, may be preserved, while lower prostaglandin production can alleviate pain and inflammation in diseases largely driven by COX-2 activity. In other words, *S. salviifolia,* which exhibits strong inhibition of COX-2 but only weak inhibition of 5-LOX, appears to selectively modulate prostaglandin-mediated inflammatory responses—such as those involved in arthritis or pain—without markedly affecting leukotriene pathways that are more commonly associated with asthma and allergic conditions.

The findings of the present study are consistent with previous research. For instance, the aqueous extract of *S. salviifolia* has been evaluated for its ligand activity on liver X receptors (LXRs), which play a crucial role in inflammation. The extract demonstrated strong agonistic activity [51]. More recently the same researchers investigated the aqueous extract, fractions, and pure compounds of *S. salviifolia*’s aerial parts in vitro and in silico, reporting that the extract reduced nitric oxide (NO) and interleukin-6 (IL-6) levels by regulating the nuclear factor kappa B (NF-κB) or hypoxia-inducible factor (HIF-1) pathways and suppressing inducible nitric oxide synthase (iNOS) [27]. The current study confirmed the in vitro COX-2 inhibitory effect of both the aerial and root extracts of *S. salviifolia.* Furthermore, a study by Şenol et al. (2010) evaluated the antioxidant effect of *S. salviifolia*’s aerial methanol extract using the DPPH radical scavenging assay, yielding an inhibition value of 52.15 ± 0.74% [21]. In comparison, the present study found the aerial part to exhibit 86.8 ± 0.28% inhibition and the root extract to show 87.9 ± 0.36% inhibition, both of which are significantly higher than the previously reported values.

Previous studies on *S. salviifolia* have revealed that the chloroform extract of *S. salviifolia* roots displays strong antifungal activity against *C. krusei*, with a minimum inhibitory concentration (MIC) of 32 μg/mL, compared to the positive control, fluconazole (MIC: 64 μg/mL) [25]. The methanol extract of *S. salviifolia* aerial parts exhibited selective cytotoxic activity against HEp-2 and HeLa cancer cell lines [23].

The methanol extract of *S. salviifolia* was reported to exert tyrosinase inhibitory and antioxidant activities; the ethyl acetate extract of the aerial parts of *S. salviifolia* exhibited AChE, BChE, and α-amylase inhibitory activities. The total phenolic and flavonoid contents were found to be the highest in the aqueous extracts and lowest in the ethyl acetate extracts [24].

Additionally, *S. salviifolia* has been reported to contain a range of secondary metabolites, including flavonoids and phenolic acid glycosides [20,22,24]. As a result of a previous phytochemical study on the methanol extract of the aerial parts of *S. salviifolia*, two new methyl-α-pyrone glucoside-type compounds, scusalvioside A and scusalvioside B, were isolated. Additionally, three phloroglucinol glucosides, namely, phlorin, tadehaginoside, and 6″-*O*-Z-p-coumaroyl phloroglucinol-1-*O-β*-glucopyranoside; six flavonoids, including apigenin, apigenin 5-*O*-*β*-glucopyranoside, isoschaftoside, luteolin 7-*O*-*β*-glucuronide, luteolin 4′-*O*-*β*-glucopyranoside, and hispidulin; and one phenylethanoid glycoside, martynoside, were also identified [52]. In the present study, we identified 29 phenolic compounds in the aerial part and root extracts; 18 of these were found to be common. Significant anti-inflammatory and antioxidant properties were previously demonstrated by the compounds coumaric acid, ferulic acid, apigenin, quercetin, chrysin, wogonin, kaempferol, baicalein, and naringenin and their glycosides. Through pathways such as NF-κB and mitogen-activated protein kinase (MAPK), these constituents modulate various inflammatory processes, including the suppression of pro-inflammatory cytokines and enzymes such as COX-2 [53,54,55,56,57,58]. By scavenging free radicals and lowering oxidative stress, they also exhibit potent antioxidant activities that aid in preventing cellular damage [59,60]. When taken together, these compounds may help prevent and treat disorders linked to inflammation and oxidative stress. The aforementioned secondary metabolites, with identified anti-inflammatory and antioxidant effects, have also been detected in *S. salviifolia* extracts herein. Therefore, the present study aligns with previous studies conducted on these compounds.

The literature indicates that studies on anti-inflammatory activity have been conducted on other *Scutellaria* species. The extract of *S. baicalensis* and *Acacia catechu* reduced edema in a dose-dependent way in a mouse model of arachidonic acid-induced ear swelling. In a rotary drum walking model, the combined extract reduced swelling and restored function when arachidonic acid was administered directly into the intra-articular space of mouse ankle joints. These findings imply that in an in vivo model of inflammation, this mixture reduces the synthesis of pro-inflammatory eicosanoids and attenuates edema by the “dual inhibition” of COX and LOX enzymes. However, with 50% inhibitory concentrations (IC_50_) of 15 µg/mL, this extract suppressed the activity of the COX-1 and COX-2 peroxidase enzymes. A less strong effect on 5-LOX was shown by the same extract’s higher IC_50_ of 25 µg/mL for 5-LOX enzyme activity [61]. The present study examined the enzyme inhibitory effects of the extracts at a single concentration. However, developing a concentration–response curve is essential, as it illustrates the relationship between the concentration of the test material and its biological effect, ensuring the reliability and reproducibility of the observed responses. It can contribute to a deeper understanding of a compound’s efficacy and safety profile, guiding further in vivo studies and therapeutic development. Therefore, it is intended to evaluate the activities at various concentrations and determine the IC_50_ value, necessitating the acquisition of new kits in further studies.

The medicinal properties of another *Scutellaria* species, *S. brevibracteata*, in managing inflammation were explored by Doğan et al., 2022 [62]. The study utilized network pharmacology and molecular docking analysis to uncover the underlying molecular pathways involved in inflammation. Experimental confirmation was achieved using lipopolysaccharide (LPS)-stimulated RAW 264.7 cells, as measured by the cytokines NO and IL-6. Additionally, antioxidant activity was assessed by analyzing the radical scavenging capabilities of different radicals. The analysis identified the tumor necrosis factor (TNF) signaling pathway and the HIF-1 signaling pathway as key contributors to inflammation. Furthermore, based on 26 frequent protein–protein interactions, AKT1, TNF, epidermal growth factor receptor (EGFR), and COX-2 were highlighted as priority targets. The study suggests that the anti-inflammatory effects of the extracts, fractions, and pure compounds occur either by reducing NO production through the suppression of iNOS via the HIF-1 pathway or by lowering NO and IL-6 levels through the regulation of the NF-κB pathway, in alignment with the findings from network analysis and the literature [62]. It is suggested that similar network pharmacology analyses be conducted in future studies for *S. salviifolia* as well. More to the point, to reveal the therapeutic potential of *S. salviifolia* in diseases related to oxidative stress and inflammation, further detailed antioxidant and anti-inflammatory, neuroprotective (especially on cellular models of Parkinson’s disease, such as SH-SY5Y cells), and cytotoxicity studies should be conducted on this plant. To more comprehensively elucidate the anti-inflammatory potential of this plant, future studies should investigate additional inflammation-related targets, including enzymes (e.g., COX-1, iNOS), cytokines (e.g., TNF-α, IL-1β, IL-6), transcription factors (e.g., NF-κB, STAT3, AP-1), receptors (e.g., TLRs, TNFR, IL-1R), and major signaling pathways such as NF-κB, MAPK, JAK/STAT, and PI3K/Akt, all of which play key roles in the initiation and progression of inflammatory responses.

## 4. Materials and Methods

### 4.1. Materials

*Scutellaria salviifolia* Benth. was collected from Beyşehir road in Konya. The plant material was identified by Prof. Dr. Osman TUGAY, and the herbarium specimen is stored at the Konya (KNYA) Selçuk University Herbarium (O.TUGAY 20863). The following materials were used: 5-lipoxygenase (5-LOX) (Abcam, Cambridge, UK, ab284521), cyclooxygenase-2 (COX-2) (Abcam, ab283401) enzyme kits, 2,2-azinobis(3-ethylbenzothiazoline-6-sulfonic acid) (ABTS, Sigma-Aldrich, St. Louis, MO, USA), 2,2-diphenyl-1-picrylhydrazyl (DPPH, Sigma-Aldrich), and potassium persulfate (Sigma-Aldrich). Spectrophotometric and fluorometric measurements were performed using a microplate reader (Multiskan Go, Thermo Scientific Inc.; Spectramax i3x, Molecular Devices, Waltham, MA, USA).

### 4.2. Preparation of Plant Materials and Extracts

Dried and powdered aerial and root parts of *S. salviifolia* were extracted separately using methanol (MeOH) (3 × 500 mL). The resulting extracts were concentrated and dried under reduced pressure at 40 °C and were stored in this form for subsequent studies. The extraction yields were determined as 23.14% for the aerial parts and 11.78% for the root parts.

### 4.3. Enzyme Inhibition Assays

#### 4.3.1. 5-Lipoxygenase (5-LOX) Enzyme Inhibition

The in vitro 5-LOX inhibition capacities of the extracts were determined by a “5-LOX inhibitor screening kit” (Abcam, ab284521). 5-LOX is an enzyme involved in converting unsaturated fatty acids to epoxides, such as the production of leukotrienes from arachidonic acid. This process plays a significant role in cell proliferation, differentiation, and inflammation. The method utilized a human 5-LOX enzyme and the 5-LOX inhibitor Zileuton as a positive control. Fluorescence measurements were performed at 500/536 nm (excitation/emission).

#### 4.3.2. Cyclooxygenase-2 (COX-2) Enzyme Inhibition

The in vitro COX-2 inhibition capacities of the extracts were assessed using the “Cyclooxygenase-2 Inhibitor Screening Kit” (Abcam, ab283401). The COX enzyme is involved in the biosynthesis of prostanoids, including prostaglandins, prostacyclin, and thromboxanes, from arachidonic acid. The level of COX-2 enzyme in cells significantly increases during inflammation. The method employed a human recombinant COX-2 enzyme and the selective COX-2 inhibitor Celecoxib as a positive control. Fluorescence measurements were performed at 535/587 (excitation/emission) nm.

### 4.4. Antioxidant Activity Assays

#### 4.4.1. DPPH Radical Scavenging Activity

The antioxidant activity of the plant extracts was determined by evaluating the DPPH radical scavenging activity, using the method described by Clarke et al., 2013 [63]. Test solutions, diluted with 20 μL of dimethyl sulfoxide (DMSO), were mixed with a 40 μg/mL DPPH solution prepared in 180 μL of methanol in a 96-well plate. After incubating in the dark for 15 min, the absorbance was measured at 540 nm using an ELISA reader (Multiskan SkyHigh Microplate spectrophotometer, Thermo Scientific, Waltham, MA, USA). DMSO was used as the blank instead of the test sample, and quercetin solution prepared with DMSO was used as the standard. The results are expressed as % DPPH scavenging effect using the following formula:

DPPH Scavenging Effect% = [(A_control_ − A_sample_)/(A_control_)] × 100

where A_control_ is the absorbance of the solutions without the test substances, and A_sample_ is the absorbance of the extract or quercetin.

#### 4.4.2. ABTS Radical Scavenging Activity

The ABTS radical scavenging activity of the plant extract was determined using the method of Chun et al., 2005 [64]. The test samples were prepared in the same manner as for the DPPH assay. ABTS+ radical was prepared by reacting 15 mL of 7 mM ABTS with 264 μL of 140 mM potassium persulfate solution and then keeping the mixture in the dark at room temperature for 16 h (stock solution). The ABTS working solution was adjusted to an absorbance of 0.70 ± 0.02 at 734 nm by diluting the stock solution with methanol. A 50 μL sample solution was mixed with 100 μL of the ABTS working solution in a 96-well plate. After the mixture was incubated at room temperature for 10 min, the absorbance was measured at 734 nm against the blank. The ABTS+ scavenging activity of the plant extract was compared with BHT, and the inhibition percentage was calculated using the following formula:

ABTS+ Scavenging Activity% = [(A_control_ − A_sample_)/(A_control_)] × 100

where A_control_ represents the absorbance of the ABTS+ radical and methanol mixture, and A_sample_ represents the absorbance of the ABTS+ radical and test or reference solution.

### 4.5. Phytochemical Analysis

#### Quadrupole Time of Flight Liquid Chromatography/Mass Spectrometry (Q-TOF LC/MS) Analysis Conditions

Chromatographic analyses were performed on an Agilent 1260 series High Performance Liquid Chromatography (HPLC) system coupled with an Agilent 6530 Accurate Mass Quadrupole Time-of-Flight Mass Spectrometer (Agilent Technologies, Waldbronn, Germany), equipped with a dual-spray electrospray ionization source. The HPLC system included a binary pump with an online degasser, a HiP sampler, and a column compartment (Agilent Technologies, Inc., Santa Clara, CA, USA). The separation of compounds was conducted using an Agilent Poroshell 120 SB-C18 (3 mm × 100 mm × 2.7 µm) reverse-phase column (Santa Clara, CA, USA), maintained at 35 °C. The analytes were eluted from the column using gradient elution with a mobile phase consisting of water (A) and acetonitrile (B), both containing 0.1% formic acid. The gradient elution profile was optimized as follows: 0 min, 5% B; 14 min, 20% B; 22 min, 40% B; 24 min, 70% B; 29 min, 5% B, followed by holding for 7 min. The flow rate, injection volume, and total analysis time were set to 0.6 mL/min, 5 µL, and 36 min, respectively. The MS system was operated with negative and positive electrospray ionization in a 2 GHz extended dynamic range mode. All ion modes with collision energies of 0 eV, 10 eV, 20 eV, and 40 eV were used to fragment the parent molecules of the chromatographically separated compounds and to form product ions. Full-scan MS was acquired from *m*/*z* 100 to 1000 with a scan rate of 1 spectrum/s. The ion source parameters were as follows: drying gas temperature—325 °C; drying gas flow—11 L/min; nebulizer pressure—35 psi; capillary voltage—3000 V; and fragmentor voltage—150 V. Agilent MassHunter Qualitative B.07.00 software was used for data analysis. The probabilities of the molecules overlapping with the obtained parent ion value were evaluated with fragmentation ions (product ions), and the identification processes were carried out through Metlin PCDL Manager (B.07.00) and the PubChem databases, along with published literature data.

### 4.6. Statistical Analysis

The statistical analysis was conducted using GraphPad Prism, Version 8. The enzyme inhibitory effects and antioxidant activity results were statistically analyzed using one-way ANOVA, followed by Dunnett’s multiple comparison test to compare the positive control with the experimental groups. *p* < 0.05 was accepted as statistically significant.

## 5. Conclusions

Ethnobotanical data and literature records serve as invaluable resources for identifying bioactive compounds in medicinal plants. Among the *Scutellaria* species, *S. baicalensis* and *S. barbata* are the most well-known and extensively studied ones. These species are widely used in East Asian folk medicine for their anti-inflammatory, antiviral, sedative, antithrombotic, and antioxidant properties. *S. salviifolia* is also documented in traditional practices in Türkiye. The findings of the current study suggest that *S. salviifolia* may be effective in managing inflammatory-related disorders, likely due to the presence of phenolic compounds. Inflammation, beyond its role in infection prevention, is critical in numerous physiological processes. Chronic or severe inflammation is associated with a wide range of diseases affecting various systems, such as infectious, neurodegenerative, cardiovascular, autoimmune, respiratory, metabolic, and cancerous diseases. According to the enzyme inhibitory and antioxidant effects of both the aerial parts and roots of *S. salviifolia,* this plant may serve as a valuable alternative to the most well-known *Scutellaria* species, including *S. baicalensis* and *S. barbata.* However, for medicinal use and the development of new therapeutic agents, further advanced preclinical and clinical studies are required to thoroughly analyze the plant extracts and elucidate their phytochemical content.

## Figures and Tables

**Table 1:** ABTS and DPPH radical scavenging effects and IC_50_ values of *S. salviifolia* extracts.

Extracts and References	ABTS Radical Scavenging Activity% ± S.D. ^a^ (IC_50_: μg/mL) 2 mg/mL ^b^	DPPH Radical Scavenging Activity% ± S.D. ^a^ (IC_50_: μg/mL) 2 mg/mL ^b^
*S. salviifolia* aerial part extract	89.2 ± 0.03 ^ns^ (IC_50_: 28.77 ± 0.20)	86.8 ± 0.28 * (IC_50_: 40.7 ± 1.15)
*S. salviifolia* root extract	88.6 ± 0.88 ^ns^ (IC_50_: 31.75 ± 0.15)	87.9 ± 0.36 ^ns^ (IC_50_: 40.1 ± 0.81)
References	89.5 ± 0.26 (IC_50_: 12.33 ± 0.26) ^c^	87.4 ± 0.32 (IC_50_: 3.26 ± 0.09) ^d^

^a^ Standard deviation, ^b^ Stock concentration, ^c^ BHT (250 µg/mL), ^d^ Quercetin (1 mg/mL), ^ns^ not significant, * *p* < 0.1.

**Table 2:** Phenolic compounds identified in *S. salviifolia* aerial part (herba) and root extracts by Q–TOF LC/MS.

No.	RT (min)	Precursor Ion (*m*/*z*)	Product Ion (*m*/*z*)	Ionization Mode	Proposed Compound	Reference/Database	Herba	Root
1	0.95	133.0147	115.0036, 89.0238, 71.0135	[M-H]^−^	Malic acid	L2/Metlin	✓	✓
2	1.08	191.0194	111.0088, 87.0086, 57.0342	[M-H]^−^	Citric acid	L1/Metlin		✓
3	2.69	303.0766	141.0199, 129.0195, 113.0241	[M-H]^−^	Pentahydroxyflavanone	X/Metlin	✓	✓
4	4.16	341.0891	179.0361, 161.0249, 133.0295	[M-H]^−^	Caffeoyl hexoside 1	L1/Metlin	✓	
5	4.70	341.0877	161.0245, 135.0449, 93.0347	[M-H]^−^	Caffeoyl hexoside 2	L1/Metlin	✓	
6	5.44	341.0887	179.0357, 161.0247, 135.0448	[M-H]^−^	Caffeoyl hexoside 3	L1/Metlin	✓	✓
7	5.82	165.0548	147.0442, 119.0491, 91.0543	[M+H]^+^	*p*-Coumaric	Metlin and PubChem	✓	
8	6.24	179.0349	135.0448	[M-H]^−^	Caffeic acid	LY/Metlin	✓	
9	6.37	325.0930	205.0505, 163.0398, 145.0296	[M-H]^−^	Coumaric acid-*O*-hexoside 1	L1	✓	
10	6.91	355.1049	217.0509, 193.0506, 175.0401	[M-H]^−^	Ferulic acid-*O*-hexoside	L1	✓	
11	7.10	475.1816	329.1245, 311.1120, 161.0452	[M-H]^−^	Phenylethanoid glycoside	L1		✓
12	7.25	325.0931	205.0503, 163.0398, 145.0296	[M-H]^−^	Coumaric acid-*O*-hexoside 2	L1	✓	
13	8.45	355.1037	295.0824, 265.0720, 235.0612	[M-H]^−^	Ferulic acid-8-*C*-hexoside	L1	✓	
14	8.85	163.0402	119.0497, 93.0348, 65.0391	[M-H]^−^	*m*-Coumaric	Metlin and PubChem	✓	
15	10.18	563.1419	443.0994, 383.0784, 353.0681	[M-H]^−^	Apigenin-*C*-hexoside-*C*-pentoside	L1	✓	✓
16	10.45	477.0674	301.0350	[M-H]^−^	Quercetin glucuronide isomer 1	Metlin	✓	✓
17	11.80	477.0677	301.0359	[M-H]^−^	Quercetin glucuronide isomer 2	Metlin	✓	✓
18	11.93	547.1457	457.1136, 367.0826, 337.0720	[M-H]^−^	Chrysin-6-*C*-glucoside-8-*C*-arabinoside	L1		✓
19	12.46	461.0829	285.0437	[M-H]^−^	Flavonoid-*O*-glucuronide	L1	✓	✓
20	12.87	547.1458	457.1142, 427.1036, 367.0827, 337.0721	[M-H]^−^	Chrysin-6-*C*-arabinoside-8-*C*-glucoside	A/Metlin		✓
21	12.93	431.0980	269.0467, 225.0563, 161.0249	[M-H]^−^	Apigenin-*O*-hexoside	L1	✓	✓
22	13.07	623.1985	461.1662, 269.0459, 179.0368, 161.0250	[M-H]^−^	Verbascoside	A/Metlin and PubChem	✓	✓
23	13.88	461.0796	285.0433, 113.0240	[M-H]^−^	Scutellarin	L1/PubChem	✓	✓
24	14.27	621.1621	445.1241, 283.0660, 268.0416	[M-H]^−^	Wogonin-*O*-glucuronide-*O*-hexoside	L1		✓
25	14.60	445.0781	269.0489	[M-H]^−^	Apigenin-*O*-glucuronide	L1	✓	✓
26	15.48	475.0972	299.0603, 284.0367	[M-H]^−^	Methoxylated flavonoid-*O*-glucuronide	L1	✓	✓
27	15.82	609.1266	323.0771, 285.0411, 161.0247	[M-H]^−^	Kaempferol 3-(6″-caffeoylglucoside)	Metlin	✓	
28	16.28	365.0049	285.0446, 241.0536, 213.0581	[M-H]^−^	Scutellarein derivative	L1	✓	✓
29	16.55	447.0933	271.0601, 123.0072	[M+H]^+^	Baicalin	Metlin and PubChem	✓	✓
30	16.55	891.1861	445.0865, 269.0495, 175.0262	[M-H]^−^	Apigenin-*O*-glucuronide (dimer) 1	L1		✓
31	17.02	447.1026	271.0600, 175.0261, 113.0242	[M-H]^−^	Naringenin-7-*O*-β-D-Glucuronide	Metlin		✓
32	17.11	623.1406	303.0724, 285.0405	[M-H]^−^	Luteolin 7-(6″-ferulylglucoside)	Metlin	✓	
33	17.49	891.1878	445.0867, 269.0493, 175.0284	[M-H]^−^	Apigenin-*O*-glucuronide (dimer) 2	L1		✓
34	17.82	285.0411	133.0297, 175.0273	[M-H]^−^	Luteolin	L2/Metlin	✓	✓
35	18.11	459.1016	283.0617	[M-H]^−^	Wogonin derivative	L1	✓	✓
36	18.63	459.1031	283.0607, 268.0371	[M-H]^−^	Wogonin-*O*-glucuronide	L1	✓	✓
37	18.96	489.1149	313.0715, 175.0248	[M-H]^−^	Dimethyl flavonoid-*O*-glucuronide	L1	✓	✓
38	20.44	271.0615	253.0510, 169.0135, 123.0079	[M+H]^+^	Norwogonin	A/PubChem		✓

“✓” shows the presence of the related compound.

## Data Availability

The data presented in this study are available on request from the corresponding author.

## Abbreviations

The following abbreviations are used in this manuscript:

5-LOX	5-Lipoxygenase
AA	Arachidonic acid
ABTS	2,2-Azinobis(3-ethylbenzothiazoline-6-sulfonic acid)
BHT	Butylated hydroxytoluene
COX	Cyclooxygenase
DMSO	Dimethyl sulfoxide
DPPH	2,2-Diphenyl-1-picrylhydrazyl
EGFR	Epidermal growth factor receptor
HIF-1	Hypoxia-inducible factor
HPLC	High-performance liquid chromatography
IC_50_	Half-maximal inhibitory concentration
IL	Interleukin
iNOS	Inducible nitric oxide synthase
LPS	Lipopolysaccharide
LT	Leukotriene
LXR	Liver X receptor
MAPK	Mitogen-activated protein kinase
MeOH	Methanol
MIC	Minimum inhibitory concentration
NF-κB	*N*uclear *f*actor kappa B
NO	Nitric oxide
PG	Prostaglandin
Q-TOF LC/MS	Quadrupole time of flight liquid chromatography/mass spectrometry
ROS	Reactive oxygen species
S.D.	Standard deviation
TIC	Total ion chromatogram
TNF	Tumor necrosis factor
